# The role of ESRP1 in solid tumor development through the regulation of CD44 splicing and EMT processes

**DOI:** 10.3389/fonc.2025.1451130

**Published:** 2025-02-12

**Authors:** Lili Wang, Min Zhang, Kelei Zhao, Xiaohan Yuan, Houyu Zhao, Yanting Liu, Yinghua Ji, Ping Lu

**Affiliations:** Department of Oncology, The First Affiliated Hospital of Xinxiang Medical University, Weihui, Henan, China

**Keywords:** ESRP1, CD44, EMT, alternative splicing, solid tumor

## Abstract

According to the World Health Organization’s statistics, cancer is the second leading cause of death worldwide, following cardiovascular diseases. Despite significant progress in the field of cancer treatment in recent years, cancer remains one of the main factors shortening human life expectancy. The field of cancer research is increasingly focusing on the role of tumor-related oncogenes and heterogeneous proteins in the development of cancer. Studies indicate that there is a close connection between solid tumors and epithelial splicing regulatory protein 1 (ESRP1). ESRP1 is a key intracellular molecule that plays a crucial role in cell growth and differentiation. As an emerging biomarker, ESRP1 has a decisive impact on the formation and development of solid tumors by regulating the alternative splicing of CD44 and the epithelial-mesenchymal transition (EMT) process. Research shows that abnormal expression of ESRP1 is closely related to the formation and development of various solid tumors, including breast cancer, lung cancer, stomach cancer, and others, and is closely associated with the invasiveness, metastasis, and poor prognosis of tumors. Therefore, given ESRP1’s critical role in cancer development, it is gradually becoming a potential biomarker and therapeutic target. This review primarily discusses the molecular mechanisms of ESRP1 in regulating cancer metastasis, particularly its regulatory effects on CD44 splicing and the EMT process. These research findings provide new targets for cancer treatment, aiming to bring more precise diagnosis and more effective treatment strategies to patients.

## 1 Introduction

Cancer has become a global public health and economic problem in the 21st century, accounting for approximately 1/6 (16.8%) global deaths and approximately 1/4 (22.8%) deaths from non-communicable diseases. According to the latest The Global Cancer Observatory (GLOBOCAN) estimates from the International Agency for Research on Cancer (IARC), cancer leads to a high proportion of premature deaths, especially among the 30-to-69-year-old age group, where it is a major cause of death (1). Additionally, cancer not only hinders the global increase in healthy lifespan but also imposes significant costs on society and the macroeconomy. Based on analyses of future population growth and aging, even if the overall cancer incidence remains unchanged, the global number of new cancer cases is projected to increase from 20 million in 2022 to over 35 million by 2050, a 77% increase. This growth is primarily driven by changes in population structure, with the global population expected to increase from 8 billion in 2022 to 9.7 billion by 2050 (2). Therefore, the presence of malignant tumors places a heavy economic burden and mental stress on individuals and is a challenge that the world is finding difficult to overcome. Finding effective treatment targets and methods is crucial for alleviating the health, social, and economic burden caused by cancer.

## 2 The role of ESRP1 in cancer development and progression

### 2.1 Role of ESRP in cancer development

In recent years, oncology research has been continuously deepening, especially in exploring cancer-related genes and heterogeneous proteins, which has gradually become a research hotspot. Epithelial splicing regulatory protein (ESRP) has attracted widespread attention from researchers against this backdrop. Its potential role in the field of oncology and its contribution to various types of tumors are gradually being confirmed. The latest research findings from numerous scholars indicate that ESRP not only plays a key role in the development of malignant tumors such as lung cancer (3) and breast cancer (4), but also plays an important role in complex diseases such as gastric cancer (5) and clear cell renal cell carcinoma (6), melanoma (7), oral squamous cell carcinoma (8), and other related tumors. Further research reveals that ESRP plays diverse roles in different tumors, acting either as an oncogene to promote tumor progression or as a tumor suppressor to inhibit the occurrence and development of tumors. This discovery has opened new avenues for tumor research and treatment. Overall, ESRP plays a key role in the development of various solid tumors and its dual identity as an oncogene or tumor suppressor gives it extremely high value and significance in oncology research. With the continuous deepening of ESRP research and the advancement of technology, we hope to provide new strategies and directions for the treatment of different types of tumors by regulating the expression of ESRP.

### 2.2 ESRP1 as a key regulator in invasion and metastasis

**Figure 1:**
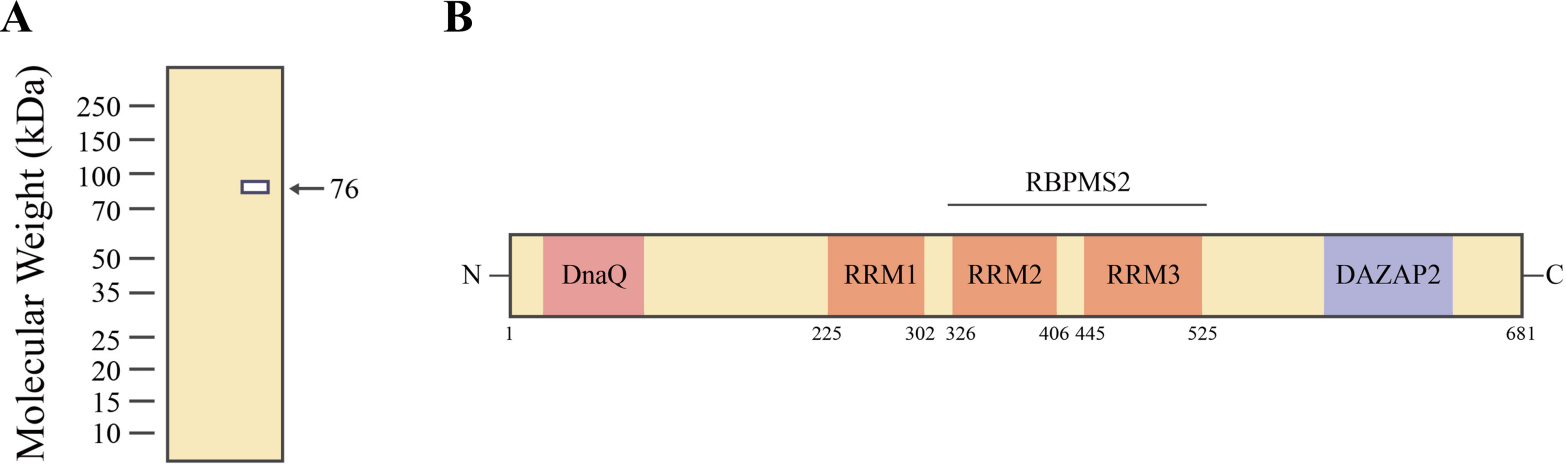
ESRP1 molecular weight and structure. **(A)** The molecular weight of ESRP1 at 76 kDa based on a Western blot experiment. **(B)** The structure of ESRP1 consists of DnaQ-like exonuclease domain at the N-terminus, followed by three conserved RNA recognition motif (RRM) domains (RRM1–3), and lastly, a proline-rich region that is homologous to DAZ-associated protein 2 at the C-terminus. RBPMS2 [multiple splicing (variants) 2] , the RRM2 and RRM3 domains of ESRP1, mediate its direct interaction with RNA-binding protein with multiple splicing-2, thereby regulating smooth muscle cell plasticity. Reproduced with authorization (18). Part of *Springer Nature* (2022).

Invasion and metastasis are significant features of cancer, closely associated with cancer-related deaths. Invasion is the first step of metastasis, which is a complex, multistep process crucial for cancer cells to spread to anatomically distant organs (9). ESRPs are widely studied alternative splicing (AS) regulatory factors (10), specifically expressed in epithelial cells (11). Recent studies have indicated that ESRPs are important regulatory factors for invasion and metastasis in the progression of cancer (12, 13). AS is a vital biological process that can generate multiple messenger RNAs (mRNAs) from a single gene (14), playing an essential role in regulating epithelial-mesenchymal transition (EMT)-related signals, cytoskeletal remodeling, tumorigenicity, and metastasis (15). Warzecha et al. conducted genome-wide screening to identify factors that promote epithelial cell splicing. Among various factors, they identified two protein homologs that induce epithelial-specific splicing patterns. Although the expression of these two genes is highly cell-type-specific, upregulation of these genes is commonly observed in epithelial cell types. These proteins were renamed epithelial splicing regulatory protein 1 and 2 (ESRP1 and ESRP2) (16). The ESRP family consists of two members with similar structure and function, namely, ESRP1 and ESRP2 (also known as RBM35A and RBM35B) and these are members of the heterogeneous nuclear ribonucleoprotein (hnRNP) family of RNA-binding proteins. The *ESRP1* gene is located on human chromosome 8q22.1, encoding a functional ESRP1 protein with an estimated molecular weight of 76 kDa (**Figure 1A**), consisting of 681 amino acids and composed of three conserved tandem recognition motifs. It is predominantly present in the nucleus, expressed in multiple organs, and detectable in the cytoplasm (**Figure 1B**). The *ESRP2* gene is located on human chromosome 16q22.1, producing a nearly 78 kDa ESRP2 protein composed of 727 amino acids (17). Although ESRP1 shares a similar primary structural organization with its paralog ESRP2, these two proteins have distinct functions in different cancers (8). In this article, we mainly focus on the various roles of ESRP1.The expression and activity of ESRP1 are regulated by various mechanisms, including post-translational modifications and non-coding RNAs (18). ESRP1 exhibits both anti-cancer and pro-cancer effects in different cancer types. Recent studies have found that ESRP1 influences cell-cell adhesion, cytoskeletal organization, and cell migration by regulating the alternative splicing of multiple genes, including *CD44*, *CTNND1*, *ENAH*, and *FGFR2* (8, 19).

### 2.3 ESRP1 as a key regulator in tumorigenesis

**Figure 2:**
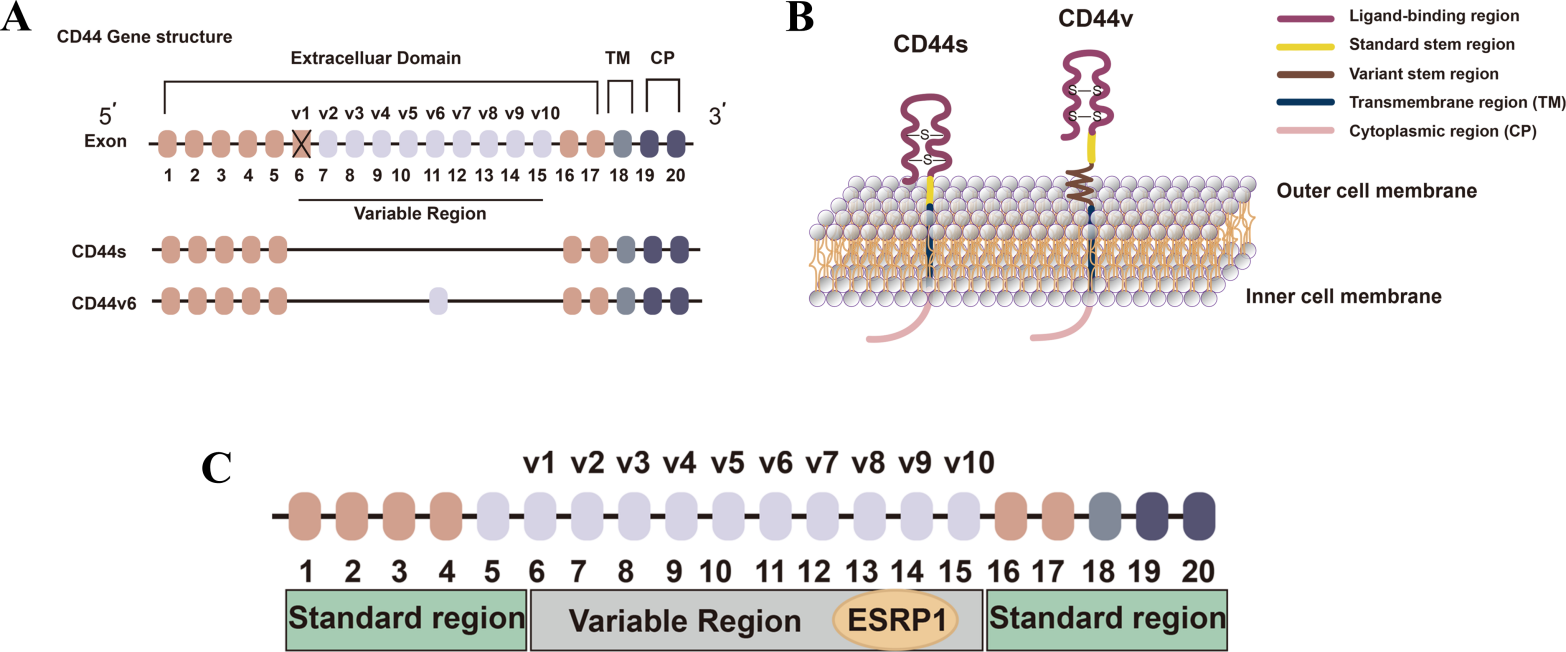
CD44 and ESRP1. **(A)** *CD44* gene structure and its related isoform structure diagram. Reproduced with authorization (26) (2021). *Federation of European Biochemical Societies.* **(B)** Protein structure of CD44 Isoforms. Reproduced with authorization (26) (2021). *Federation of European Biochemical Societies*. ***(*C*)*** The interaction between CD44 and ESRP1. Reproduced with authorization (27). Chen et al., *Journal of Hematology & Oncology* (2018) 11:64. Modified color and shape. https://creativecommons.org/licenses/by/4.0/.

CD44 is a cell surface transmembrane glycoprotein involved in various cellular processes, including cell division, survival, migration, and adhesion. Since the discovery of cancer stem cells (CSCs) in solid tumors, CD44 has been widely used as a CSC marker in breast cancer (20) and other malignancies. The human *CD44* gene is located on chromosome 11p13 and is composed of 19 exons, with 10 constitutive exons present in all isoforms. The standard form of CD44 (CD44s) is encoded by these constitutive exons. CD44 variant isoforms (CD44v) arise from selective splicing, combining the 10 constitutive exons with the remaining nine variant exons. Selective splicing mediated by ESRPs encodes a polymorphic protein group (85-250 kDa in size) (21, 22). CD44s comprises only constitutive exons, while variant CD44v isoforms contain one or more variant exons (23) (**Figure 2**). CD44 has been identified as a marker for cancer stem cells and a key factor in cancer development (24, 25).

Studies have shown that the expression level of CD44s is positively correlated with the tumor stem cell (CSC) characteristics and the invasive phenotype of breast cancer, while CD44v is negatively correlated with these features but positively correlated with cancer cell proliferation (28). Additionally, different CD44 splice variants play crucial roles in cell adhesion, migration, and signal transduction. ESRP1, by regulating the expression of CD44 splice variants, can influence the behavior of tumor cells, significantly impacting the EMT process and breast cancer metastasis (29). CD44s is associated with cell migration and invasion, while CD44v variants are associated with cell proliferation, survival, and cancer stem cell properties. Low expression of ESRP1 leads to an increase in CD44s levels and a decrease in CD44v (variant CD44) levels, both of which play crucial roles in tumor development (30). Studies have found that the transition from CD44 variants containing variable exons (CD44v) to CD44 standard isoforms (CD44s) lacking variable exons, regulated by ESRP1, is critical for the EMT process in breast cells (27, 28). Additionally, CD44 is involved in regulating the tumor microenvironment and interacts with various signaling pathways, such as Wnt, TGF-β, and EGF pathways. ESRP1 can indirectly affect the activity of these pathways by regulating the expression of CD44 splice variants, thereby influencing tumor growth, invasion, and drug resistance (29, 30).

Therefore, ESRP1 has a significant impact on tumor occurrence, development, invasion, and metastasis by regulating the alternative splicing of CD44. Modulating the splicing of CD44 could inhibit tumor invasion and metastasis, making ESRP1 or specific CD44 splice variants potential targets for cancer therapy. Further research on the relationship and mechanisms of action between ESRP1 and CD44 in cancer could lead to the development of new diagnostic markers and treatment targets.

### 2.4 ESRP1 as a key regulator in EMT

Cancer metastasis is associated with EMT. ESRP1 has been confirmed as a core regulator of EMT-related splicing events in human tumor metastasis (31, 32). It can regulate selective splicing during EMT, affecting activities such as EMT cell motility, cytoskeletal dynamics, and cell-cell adhesion (14) as well as regulating cancer cell proliferation (33) and differentiation (34, 35), playing a role in EMT during cancer progression (36).

EMT is characterized by the upregulation of mesenchymal markers (such as vimentin and N-cadherin) and the downregulation of epithelial markers (such as E-cadherin), leading to the acquisition of invasive and migratory capabilities with a mesenchymal phenotype (37). It is a fundamental process of cell shape change in animal development and disease progression (38), referring to the biological process where epithelial cells undergo a specific program to transform into mesenchymal cells. This process involves multiple biochemical changes in static and polarized epithelial cells’ lateral connections, which often involve reshaping surrounding tissues with cells such as cancer-associated fibroblasts (CAFs) and tumor-associated macrophages (TAMs) to promote angiogenesis and cell migration (39), thereby disrupting the cohesive structural integrity between epithelial cells and between cells and the extracellular matrix. This characteristic enables these cells to acquire motility and is also an important cytological basis for the invasion and metastasis of malignant tumors (40), playing a key role in the invasion and metastasis of malignant epithelial tumors. Depending on the type of disease, EMT can be roughly divided into three types: Type I EMT, which mainly occurs in organ formation and embryonic development; Type II EMT, which is associated with inflammation, wound healing, organ fibrosis, tissue repair, and regeneration, contributing to tissue reconstruction after injury (41); and Type III EMT, which is related to tumor invasion and metastasis and closely related to tumor occurrence (42). There is increasing evidence that EMT is aberrantly activated in cancer cells, playing a crucial role in mediating tumor recurrence and metastasis (43). Several excellent reviews have shown that inhibiting EMT-related pathways can suppress the development of colorectal cancer (28), gastric cancer (5), ovarian cancer (44), lung cancer (45), and prostate cancer (46), among others.

**Figure 3:**
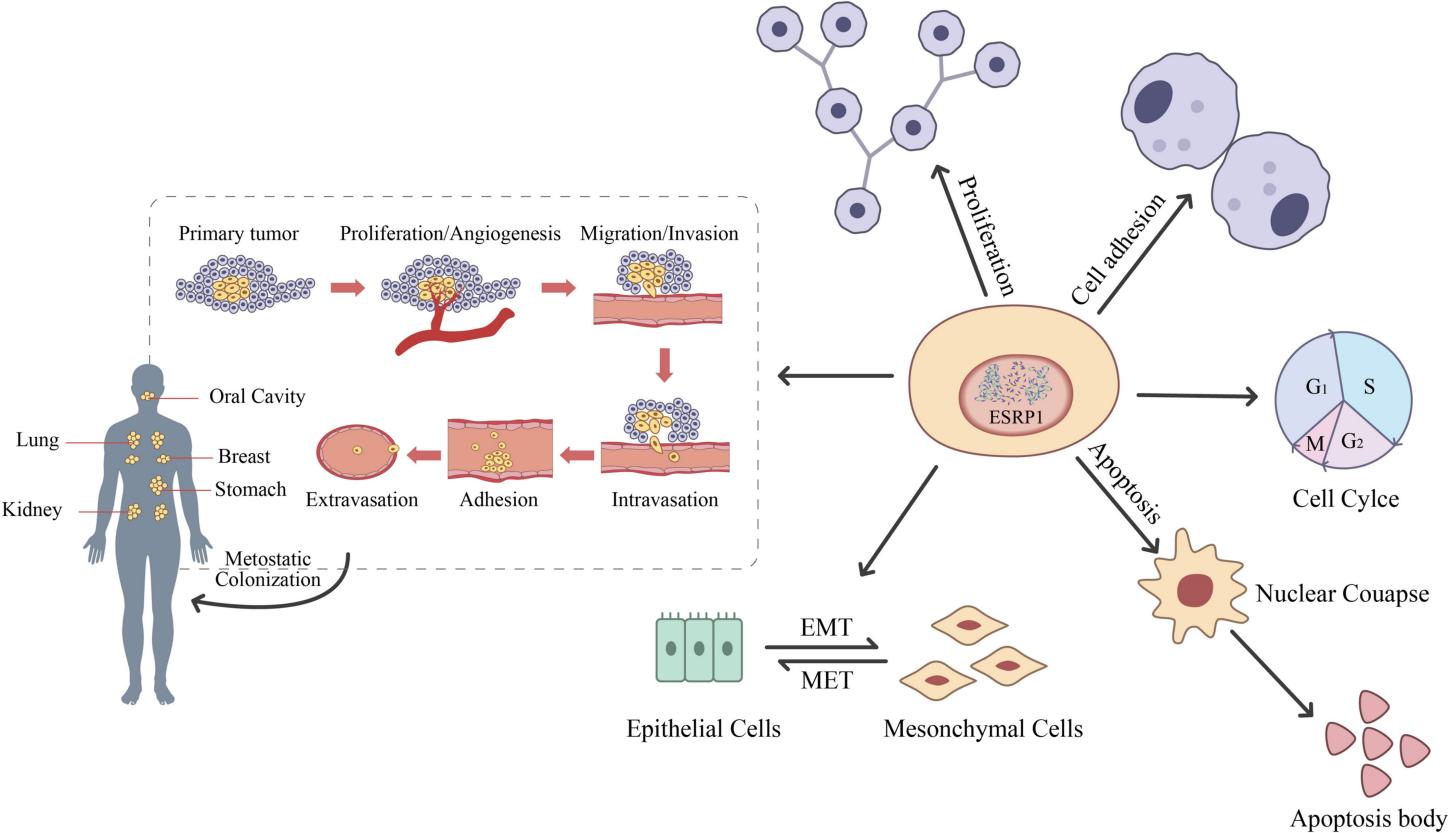
Flowchart of ESRP1-related functions. Reproduced with authorization (18). Part of *Springer Nature* (2022).

More and more evidence suggests that the dysregulation of ESRP1 is closely related to cancer progression, providing a new entry point for the treatment of cancer patients based on ESRP1 dysregulation. Therefore, in this review, we summarize the structure, function, and related pathways of ESRP1 (**Figure 3**), focusing on its potential mechanisms in the occurrence of various solid tumors (**Table 1**) and its clinical significance as a prognostic biomarker and therapeutic target for cancer patients. The information reviewed in this article may be very beneficial for the development of personalized treatment strategies for cancer patients.

**Table 1:** Role of ESRP1 in different types of cancer.

Cancer type	The function of ESRP1	Reference
ADC (Adenocarcinoma)	ESRP1 overexpression is negatively correlated with metastasis, tumor size, and clinical Stage in ADC patients.	(13)
	ESRP1 upregulates ISG15 via CREB, suppressing EMT and further inhibiting ADC progression.	(45)
SCLC (Small cell lung cancer)	ESRP1 suppresses TGF-β/Smad signaling by regulating CARM1 splicing, reversing chemoresistance in SCLC and enhancing its chemosensitivity.	(47)
GC (Gastric cancer)	ESRP1 binds to lncRNA CCAT2, which regulates CD44 splicing, promoting the transition from standard CD44 to CD44v6 and facilitating gastric cancer progression.	(42)
	Copy number gains in chromosome regions 8q22 (including ESRP1 and CCNE2),8q24 (including MYC and TNFRSF11B), and 20q11-q13 (including SRC, MMP9, and CSE1L) are associated with poorer survival in patients.	(45)
	LRRFIP2 expression is higher in high ESRP1-expressing cell lines, while LRRFIP variant 3 is higher in low ESRP1-expressing cell lines, determining gastric cancer cell metastasis via differential protein interaction with methyltransferase CARM1.	(5)
ccRCC (Clear cell renal cell carcinoma)	CircAKT3 competitively binds to miR-296-3p, leading to the upregulation of E-cadherin and suppression of ccRCC metastasis.	(48)
	ESRP1 suppresses ccRCC cell proliferation and migration by participating in the biological formation of circ-TNPO3.	(49)
	Overexpression of circESRP1 suppresses ccRCC progression *in vivo* and *in vitro* through the CircESRP1/miR-3942/CTCF axis-mediated downregulation of the c-Myc and EMT pathway.	(50)
CMM (Cutaneous malignant melanoma)	Hyaluronic acid (HA) activation of CD44 enhances the migration of melanoma and other tumor cells.	(51)
	Overexpression of CD44v promotes tumor progression.	(52)
	CD44v6 overexpression enhances melanoma brain metastasis cell migration.	(53)
	ESRP1 significantly inhibits the growth of tamoxifen-resistant cells by affecting kinase metabolism involved in mitosis, cell cycle, and proliferation, altering epithelial-mesenchymal transition protein markers.	(54)
OSCC (Oral squamous cell carcinoma)	CircRNA_100290 is upregulated in OSCC tissues, promoting OSCC progression through the circRNA 100290/miR-29b/CDK6 Axis.	(55)
	The cirhrf1/miR526b-5p/c-Myc/TGF-β1/ESRP1 pathway indicates ESRP1 promotes OSCC EMT progression.	(56)
	ZEB1/2 promotes OSCC cell invasion by suppressing ESRP1 and ESRP2-mediated selective splicing and isoform conversion of FGFRs.	(57)
BC (Breast cancer)	ESRP1 promotes tumor progression in ER+ breast cancer cells by regulating genes involved in fatty acid/lipid metabolism and oxidative reduction processes.	(58)
	Hypoxia-driven ESRP1 deletion induces skipping of hMENA exon 11a, producing the pro-migratory isoform hMENAΔ11a, which promotes breast cancer EMT through the TGF- RBFOX2-ESRP1 axis.	(59)
	Hypoxia-driven ESRP1 deletion induces skipping of hMENA Exon 11a, producing the pro-migratory isoform hMENAΔ11a, which promotes breast cancer EMT through the TGF- RBFOX2-ESRP1 axis.	(60)

## 3 The role of ESRP1 in various types of cancer

### 3.1 ESRP1 in lung cancer

ESRP1 can affect the splicing patterns of multiple genes related to lung cancer. Its regulation of CD44 alternative splicing plays an important role in the migration and invasion of lung cancer cells. Clinical studies by Cui et al. have shown that ESRP1 expression is significantly increased in precancerous lesions and non-small cell lung cancer (NSCLC) tissues compared to normal lung tissues. Moreover, high expression of ESRP1 is associated with a favorable prognosis in NSCLC patients (3). The expression and function of ESRP1 may vary in different types of lung cancer. Li et al. found that ESRP1 overexpression was negatively correlated with the presence of metastasis, tumor size, and clinical stage in lung adenocarcinoma (ADC) patients through immunohistochemical analysis of 125 clinical tissue samples of lung ADC (13). EMT is a key process by which lung cancer cells acquire migratory and invasive capabilities, and a high expression of ESRP1 is usually associated with the inhibition of EMT and the maintenance of cell epithelial properties. ESRP1 inhibits the EMT process by regulating the splicing of EMT-related genes, thereby reducing the migration and invasion of lung cancer cells.

Qu et al. demonstrated experimentally that ESRP1 upregulates ISG15 expression via CREB, inhibiting EMT in lung ADC and thus suppressing the progression of lung ADC (45). Small cell lung cancer (SCLC) is the most malignant type of lung cancer and is difficult to treat. Meng et al. found that, compared with chemosensitive cells, ESRP1 was significantly downregulated in chemoresistant cells in SCLC. The study showed that ESRP1 reverses the chemoresistance of SCLC by regulating the selective splicing of coactivator-associated arginine methyltransferase 1 (CARM1) to inhibit the TGF-β/Smad signaling pathway, thereby enhancing its chemosensitivity (47).

Due to the correlation between ESRP1 expression in lung cancer and prognosis, it has potential as a therapeutic target. Targeting ESRP1 or its splicing events can affect the behavior of tumor cells and the tumor microenvironment, thereby affecting the occurrence, development, invasion, and metastasis of lung cancer. Developing new treatment strategies, such as restoring ESRP1 expression to inhibit EMT and tumor cell invasion or reducing tumor proliferation and metastasis through specific targeting of ESRP1-regulated splicing variants, is possible. A deeper understanding of ESRP1’s specific mechanisms in lung cancer may pave the way for new diagnostic and therapeutic approaches. Therefore, the splicing factor ESRP1 can serve as a new marker molecule for drug resistance and a potential therapeutic target for patients with malignant lung tumors.

### 3.2 ESRP1 in gastric cancer

Studies have shown that ESRP1 is overexpressed in gastric cancer (GC). Deng et al. reported that lncRNA CCAT2 is overexpressed in GC tissues compared to adjacent normal tissues and is associated with poor prognosis in patients. Through experimental validation, it was found that cells overexpressing lncRNA CCAT2 promote the transformation of CD44 from its standard form to variant CD44 isoform 6 (CD44v6) by regulating the alternative splicing of CD44, and a high expression of CD44v6 is associated with more severe lymph node invasion, later T staging, and worse prognosis in gastric cancer patients. The experimental results suggest that lncRNA CCAT2 upregulates the expression of CD44v6 by binding to ESRP1, thereby mediating the selective splicing of CD44 and promoting the development of gastric cancer, which is associated with shorter patient survival (61). Many factors can affect the prognosis of gastric cancer patients and DNA copy number amplification is an important driver of solid tumors, including gastric cancer (62, 63). High-throughput whole-genome analysis of DNA copy number variations (CNVs) has identified recurrent amplification regions in GC. Additionally, these regions contain key oncogenes involved in GC progression, including MYC on 8q, SRC and MMP9 on 20q, ERBB2 on 17q, EGFR on 7p, FGFR1 on 8p, and FGFR2 on 10q (61, 64). Local amplification of these regions and the increased frequency of amplification throughout the genome are also associated with aggressive clinicopathological features and poor disease prognosis. Wang et al. found through global correlation analysis of array-based comparative genomic hybridization (aCGH) data with clinical pathological information of GC from a large cohort and further validation using a large independent cohort, that copy number gains in three chromosomal regions, 8q22 (including ESRP1 and CCNE2), 8q24 (including MYC and TNFRSF11B), and 20q11-q13 (including SRC, MMP9, and CSE1L), lead to poorer patient survival. The study revealed that the increased copy number of MYC and TNFRSF11B located on 8q24 is associated with the survival rate of GC, especially non-cardiac GC (64). Lee et al. found that the relative frequency of LRRFIP2 alternative splicing is significantly correlated with the expression level of ESRP1 in human gastric cancer cell lines and gastric cancer patient tissues. LRRFIP2 is believed to be a binding partner of flightless-1 and has been found to regulate the Wnt signal by interacting with Dvl in African clawed frog embryos, and to inhibit the activation of NLRP3 inflammasome by recruiting the Caspase-1 inhibitor Flightless-I (48, 65). The expression of the two isoforms of LRRFIP2 is highly dependent on the expression of ESRP1, with high expression in cell lines with high ESRP1 expression and high expression of LRRFIP variant 3 in cell lines with low ESRP1 expression. The fate of gastric cancer cells is determined by the differential interaction of LRRFIP2 with the methyltransferase protein CARM1 (5).

The expression level of ESRP1 may be related to prognosis in gastric cancer, with its overexpression possibly associated with malignancy and poor prognosis in gastric cancer, while its low expression may be associated with improved prognosis in gastric cancer. These findings suggest that ESRP1 may be a potential target for the treatment and prognosis of gastric cancer.

### 3.3 ESRP1 in clear cell renal cell carcinoma

Abnormal expression of circular RNAs (circRNAs) has been linked to tumorigenesis (49, 66, 67). For example, Xue et al. found that circAKT3 competitively binds to miR-296-3p, leading to upregulation of E-cadherin, thereby inhibiting clear cell renal cell carcinoma (ccRCC) metastasis (68).

RNA-binding motif enrichment analysis indicates that mRNA-binding proteins ESRP1, ESRP2, RBFOX2, and QKI regulate the selective splicing of mRNA during human kidney development (6). Circular RNAs (circRNAs) are covalently closed continuous loops formed by back-splicing, most of which originate from exons or introns of precursor mRNA (pre-mRNA) (69). High expression of ESRP1 is usually associated with the suppression of EMT and the maintenance of cell epithelial characteristics. Gong et al. reported that overexpression of circESRP1 downregulates c-Myc, a crucial oncogene involved in tumor development, through the CTCF-dependent positive feedback loop circESRP1/miR-3942/CTCF-mediated EMT pathway, thereby reducing the migration and invasion capabilities of ccRCC cells and inhibiting the progression of clear cell renal cell carcinoma both *in vitro* and *in vivo* (70).

Alternative splicing is a key mechanism that provides functional diversity in eukaryotic genomes, and abnormal splicing is associated with many human diseases, including cancer (71). Fibroblast growth factor receptor 2 (FGFR2) belongs to the transmembrane receptor tyrosine kinase family (designated FGFR1-4) and is involved in the regulation of cell proliferation, differentiation, migration, wound healing, and angiogenesis during development and adulthood (72). It has two subtypes, the IIIb subtype expressed in epithelial cells and the IIIc subtype expressed in mesenchymal cells, leading to ligand binding specificity changes. RNA-binding proteins ESRP1 and ESRP2 are known splicing factors that regulate FGFR2 IIIb splicing through these cis-elements. Ectopic expression of ESRP1 or ESRP2 is sufficient to induce subtype switching from mesenchymal IIIc to epithelial IIIb (16). Zhao et al. found that in approximately 90% of ccRCC cases, the FGFR2 subtype switches from the normal epithelial “IIIb” subtype to the mesenchymal “IIIc” subtype, which is kidney-specific, as it is rarely observed in other cancers. The FGFR2-IIIc isoform is a promising candidate biomarker for early detection, diagnosis, and targeted therapy in ccRCC (73). Pan et al. showed that ESRP1 may be involved in the biogenesis of circ-TNPO3 by targeting the flanking introns, inhibiting the proliferation and migration of ccRCC cells (54).

In conclusion, ESRP1 plays a crucial role in the development and progression of ccRCC. By regulating the selective splicing of genes, inhibiting the EMT process, and thereby affecting the interaction between cells and the microenvironment, it has a significant impact on the occurrence, development, invasion, and metastasis of clear cell renal cell carcinoma. Therefore, further research on the specific role of ESRP1 in ccRCC and the feasibility of its targeted strategies will help reveal the pathogenesis of ccRCC and provide new insights for personalized cancer therapy.

### 3.4 ESRP1 in malignant melanoma

Malignant melanoma is an aggressive skin cancer that originates from melanocytes (74). In recent years, its incidence has been steadily increasing worldwide. According to GLOBOCAN 2021 data, there are over 320,000 new cases of cutaneous malignant melanoma (CMM) annually, and over 50,000 people die from the disease each year, posing a serious threat to human health (75). In the early stages, malignant melanoma can be treated with surgical excision but this cancer is prone to metastasis (76). Metastasis and invasion are the main factors leading to recurrence and death in patients, with poor prognosis. The 5-year survival rate for stage IV patients in China is only 4.6% (77). Although the emergence and application of immune checkpoint inhibitors have improved patient survival rates, their use is often limited in the clinic due to immune-related side effects and poor drug tolerance. Related studies indicate that the metastasis of malignant melanoma is closely associated with EMT (78, 79). Therefore, enhancing the immune response, studying the immune phenotype and characteristics of the tumor microenvironment, and inhibiting or blocking the occurrence of EMT in tumor cells are expected to become one of the important targets for suppressing the invasive behavior of tumor cells.

Yao et al. found that the expression of ESRP1, an EMT splicing regulator, is negatively correlated with tumor-associated immune cell cytotoxicity in various types of tumors, suggesting a link between the EMT status of tumors and the activity of infiltrating lymphocytes. Their research demonstrated that melanoma with lower ESRP1 expression, which is associated with higher immune cell cytotoxicity, is more likely to respond to immune checkpoint blockade therapy (55). Wang et al. showed that ESRP1 in melanoma significantly inhibits the growth of tamoxifen-resistant cells by affecting kinases that regulate mitosis (56, 80), the cell cycle, and cell proliferation, thereby altering epithelial-mesenchymal transition protein markers (4). Furthermore, through experiments, they found that compared to normal tissue, ESRP1 is underexpressed in CMM tissue, and patients with low ESRP1 expression have relatively better overall survival and prognosis (7). Ichikawa et al. demonstrated that hyaluronic acid (HA) activation of CD44 leads to enhanced migration of melanoma and other tumor cells (51), Raso et al. indicated that, in melanoma, *in vivo* and *in vitro* models show that the expression of specific CD44v isoforms is associated with tumor progression (52). Marzese et al. found that CD44v6 overexpression enhances the migration of melanoma brain metastatic cells (53). These results suggest that high expression of ESRP1 promotes the development of melanoma.

In summary, the mechanisms and potential therapeutic value of ESRP1 in melanoma require further investigation. Understanding the specific role of ESRP1 in melanoma may help reveal the molecular mechanisms underlying melanoma occurrence and development, providing new insights for future treatment strategies.

### 3.5 ESRP1 in oral squamous cell carcinoma

Several circular RNAs (circRNAs) have been reported to regulate the occurrence of oral squamous cell carcinoma (OSCC). For example, research has shown that circRNA_100290 is highly expressed in OSCC tissues and promotes the progression of OSCC through the circ-RNA_100290/miR-29b/CDK6 positive pathway (81). RNA-binding proteins (RBPs) have been shown to interact with RNA and regulate gene expression, and they can also interact with circRNAs (82). ESRP1 affects the function and phenotype of OSCC cells by regulating the splicing of multiple genes related to cell proliferation, migration, and invasion. For example, downregulation of ESRP1 may lead to alternative splicing changes in E-cadherin, promoting reduced cell adhesion and increased invasiveness (8). Previous studies have indicated that ESRP1 regulates selective splicing events associated with the epithelial phenotype in OSCC (10, 57). Zhao et al. demonstrated that ESRP1 promotes the progression of OSCC EMT through the cirhrf1/miR526b-5p/c-Myc/TGF-β1/ESRP1 pathway (83). Osada et al. demonstrated that in OSCC, high expression of ZEB1/2 and low expression of E-cadherin and ESRP1/2 in OSCC cells are associated with invasive behavior and poor prognosis (57).

In conclusion, ESRP1 plays an important role in OSCC by regulating the alternative splicing of genes and affecting pathways such as EMT in tumor cells and the tumor microenvironment. Further research on the mechanism of action of ESRP1 in OSCC may help reveal the pathogenesis of this tumor and provide new targets and strategies for the treatment of OSCC.

### 3.6 ESRP1 in breast cancer

Breast cancer is a common malignant tumor, and its pathogenesis and progression involve numerous complex biological processes. Recent studies have shown that the expression level of ESRP1 is closely related to the occurrence, development, and prognosis of breast cancer (58, 59). Therefore, an in-depth exploration of the mechanism of action of ESRP1 in breast cancer and the development of targeted therapeutic drugs against ESRP1 are expected to provide new perspectives and strategies for the diagnosis, treatment, and prognosis of breast cancer.

Liu et al. have experimentally demonstrated that compared to non-breast cancer patients, the mRNA expression level of ESRP1 is significantly upregulated in breast cancer tissue samples, while the mRNA expression level of ESRP2 is not upregulated. They showed that ESRP1 overexpression enhances cancer cell proliferation and is associated with poor prognosis in BC patients, whereas ESRP2 is not (11). EMT is a dynamic, reversible process that may only occur in some cells or specific regions of tumor tissue (58, 84). EMT, when abnormally activated in cancer cells, can mediate tumor recurrence and distant metastasis (60, 85). RBFOX2 plays a dual role, acting as an important co-regulator of ESRP1 in epithelial phenotype cells and as an interstitial-specific splicing factor in more invasive breast cancer subtypes (86, 87). Fici et al. have shown through their research that during EMT the proportion between ESRP1 and RBFOX2 is significantly reduced and is positively correlated with EMT-specific phenotypes in cell models, with a good prognosis in patients (88). Pan et al. demonstrated through their study that miR-337-3p can inhibit the migration and invasion of breast cancer cells by downregulating ESRP1 (89). Gökmen et al. have shown that ESRP1 affects tumor progression in ER+ breast cancer cells by regulating genes involved in fatty acid/lipid metabolism and oxidation-reduction processes, and that high expression of ESRP1 is associated with poor prognosis in estrogen receptor-positive (ER+) breast tumors (90). Zhao et al. (91) reported a significant upregulation of ESRP1 mRNA in invasive ductal breast cancer, while Richardson et al. (92) found a significant upregulation of ESRP1 mRNA in ductal breast cancer. Ahuja et al. demonstrated experimentally that the loss of the hypoxia-driven splicing regulator ESRP1 leads to the skipping of hMENA exon 11a, resulting in the production of the pro-metastatic isoform hMENAΔ11a, which promotes EMT through the TGF- RBFOX2-ESRP1 axis and facilitates breast cancer metastasis (93). Kikuchi et al. suggested through their research that the ZEB1-RAB25/ESRP1 pathway may be involved in EMT and chemoresistance in breast cancer (94). EMT and its associated stem cell-like phenotype are considered major causes of breast cancer resistance (60, 95), especially the EMT activator ZEB1, which has been shown to have stemness and therapy resistance (96). One study reported that the overexpression of the miR-200 family is associated with an increased risk of breast cancer metastasis and promotes metastatic colonization in mouse models (97). Clinical observations show that metastatic tumors are of epithelial type (98, 99). Preca et al. found that HA is a major ECM glycoprotein polysaccharide enriched in breast tumors, supporting EMT and cooperating with CD44s to enhance ZEB1 expression. In breast cancer cell lines, HA is mainly synthesized by HAS2, which has been shown to be associated with cancer progression (100). Brown et al. demonstrated that during EMT, the expression of CD44 shifts from variant isoforms (CD44v) to standard isoforms (CD44s), and the regulation of CD44 selective splicing leads to EMT and breast cancer progression (101).

Overall, the role of ESRP1 in breast cancer is mainly through the regulation of gene splicing, affecting the behavior of tumor cells and the tumor microenvironment, thereby influencing the occurrence, development, invasion, and metastasis of breast cancer. Understanding the specific mechanism of ESRP1 in breast cancer may help develop new diagnostic and therapeutic approaches.

## 4 Discussion

Findings from previous studies are consistent with the observation that ESRP1 plays a crucial role in regulating genes associated with EMT, such as CD44 (36). However, there is conflicting evidence regarding the role of ESRP1 in different tumor types. While ESRP1 inhibits invasion and metastasis in some cancers, it may promote disease progression in others (5, 13, 49). These differences are most likely due to tumor type-specific signaling networks or interactions with the tumor microenvironment, highlighting the need for further specific studies. Understanding these dynamics is therefore critical to optimizing treatment for ESRP1.

ESRP1 is a key factor in the regulation of CD44 gene alternative splicing. By regulating the alternative splicing of CD44 and affecting its variant balance, it can regulate the adhesion, migration, signaling, and EMT processes of tumor cells, which has a significant impact on the occurrence and development of tumors (15). In addition, by regulating the expression of the CD44 variant, ESRP1 can also influence critical signaling pathways such as PI3K/AKT and Wnt/β-catenin, thereby participating in the regulation of the tumor microenvironment (102, 103). Consistent with previous studies, the expression levels of ESRP1 and CD44, and the proportion of their splicing variants, have potential value as prognostic biomarkers for multiple cancers. To address unanswered questions, future research could focus on the following: employing high-throughput RNA sequencing to comprehensively identify splicing events regulated by ESRP1 in a variety of tumor types; using CRISPR/Cas9 (104) to modulate ESRP1 expression and directly test its role in tumor progression, metastasis, and invasion; and investigating the interaction of ESRP1 with immune cells in the tumor microenvironment to assess its potential impact on immunotherapy efficacy.

Integrating these findings into a broader clinical and therapeutic context, ESRP1 becomes a promising therapeutic target for the treatment of solid tumors and may be a potential biomarker for patient prognosis. However, challenges remain, including the risk of unexpected side effects that can arise in complex splicing networks. In addition, the differential expression of ESRP1 across tumor types suggests that its therapeutic potential is likely to be study-specific, requiring tailored approaches for specific cancer types. Future advances in ESRP1 research may pave the way for more precise diagnostic and therapeutic strategies, including the development of small molecule or RNA-based interventions that target splicing events regulated by ESRP1.
